# Direct Lateral Corpectomy and Reconstruction Using an Expandable Cage Improves Local Kyphosis but Not Global Sagittal Alignment

**DOI:** 10.3390/jcm10174012

**Published:** 2021-09-05

**Authors:** Hidetomi Terai, Shinji Takahashi, Hiroyuki Yasuda, Sadahiko Konishi, Takafumi Maeno, Hiroshi Kono, Akira Matsumura, Takashi Namikawa, Minori Kato, Masatoshi Hoshino, Koji Tamai, Hiromitsu Toyoda, Akinobu Suzuki, Hiroaki Nakamura

**Affiliations:** 1 Department of Orthopaedic Surgery, Osaka City University Graduate School of Medicine, Osaka 545-8585, Japan; hterai@med.osaka-cu.ac.jp (H.T.); hoshino717@gmail.com (M.H.); koji.tamai.707@gmail.com (K.T.); h-toyoda@msic.med.osaka-cu.ac.jp (H.T.); a-suzuki@msic.med.osaka-cu.ac.jp (A.S.); hnakamura@med.osaka-cu.ac.jp (H.N.); 2 Department of Orthopaedic Surgery, Osaka General Hospital of West Japan Railway Company, Osaka 545-0053, Japan; hiroyuki19780728@yahoo.co.jp (H.Y.); m1378921@med.osaka-cu.ac.jp (S.K.); 3 Department of Orthopaedic Surgery, Ishikiri Seiki Hospital, Osaka 579-8026, Japan; dzm02716@nifty.ne.jp (T.M.); hiroshikishikiri@gmail.com (H.K.); 4 Department of Orthopaedic Surgery, Osaka City General Hospital, Osaka 534-0021, Japan; amatsumura@med.osaka-cu.ac.jp (A.M.); namikawa@msic.med.osaka-cu.ac.jp (T.N.); minori202048@gmail.com (M.K.)

**Keywords:** direct lateral corpectomy, expandable cage, global alignment, local kyphosis, osteoporosis vertebral fracture

## Abstract

Recently, an expandable cage equipped with rectangular footplates has been used for anterior vertebral replacement in osteoporotic vertebral fracture (OVF). However, the postoperative changes in global alignment have not been elucidated. The purpose of this study was to evaluate local and global spinal alignment after anterior and posterior spinal fixation (APSF) using an expandable cage in elderly OVF patients. This retrospective multicenter review assessed 54 consecutive patients who underwent APSF for OVF. Clinical outcomes were compared between postoperative sagittal vertical axis (SVA) > 95 mm and ≤95 mm groups to investigate the impact of malalignment. SVA improved by only 18.7 mm (from 111.8 mm to 93.1 mm). VAS score of back pain at final follow-up was significantly higher in patients with SVA > 95 mm than SVA ≤ 95 mm (42.4 vs. 22.6, *p* = 0.007). Adjacent vertebral fracture after surgery was significantly more frequent in the SVA > 95 mm (37% vs. 11%, *p* = 0.038). Multiple logistic regression showed significantly increased OR for developing adjacent vertebral fracture (OR = 4.76, 95% CI 1.10–20.58). APSF using the newly developed cage improves local kyphotic angle but not SVA. The main cause for the spinal malalignment after surgery was postoperative development of adjacent vertebral fractures.

## 1. Introduction

Maintenance of global sagittal balance in the standing position is important for minimizing energy expenditure and load on the musculoskeletal system [1]. Many mechanisms work together to maintain balance in the normal spine and extremities, including some compensatory mechanisms. However, once the compensatory mechanisms break down, there is severe deterioration in the patient’s condition, pain, and reduction of quality of life (QOL) [2]. Other reports have shown that osteoporotic vertebral fracture (OVF) is strongly related to sagittal spinal imbalance in aged patients [3,4,5]. Several reports suggest that reduced muscle volume (i.e., sarcopenia) is one of the major causes of sagittal imbalance, causing reduction in the QOL of OVF patients [6,7,8]. Sarcopenia and osteoporosis show a high prevalence in old age and incur a high risk for falls, fractures, and further functional decline [9]. The term osteosarcopenia has been proposed to describe individuals suffering from both diseases [10]. With the aging of society and the associated increase in the amount of osteosarcopenia [11], the number of patients presenting with problems associated with an imbalanced sagittal spine is also likely to increase in the near future.

OVF mainly occurs at the thoracolumbar junction and negatively affects spinal alignment and QOL [5]. There are many surgical methods for the treatment of OVF, such as vertebroplasty (VP), balloon kyphoplasty (BKP), anterior vertebral replacement and posterior spinal fixation (APSF), and posterior osteotomy (PO) including posterior vertebral column resection (pVCR) [12,13]. The choice of surgical method is based on the goal of surgery, the patients’ symptoms, the degree of deformity, the global spinal alignment, and the flexibility. However, few reports have described the correlation between local kyphotic changes and changes in global alignment after OVF surgery.

Recently, a newly developed expandable cage equipped with rectangular footplates has overcome the subsidence that is thought to be a disadvantage of anterior surgery for OVF. In addition, recent advances in the lateral approach enable minimally invasive anterior spinal reconstruction of thoracolumbar and lumbar lesions in elderly patients. Taiji et al. in a cohort of 16 OVF patients treated with the wide-foot-plate expandable cage reported a 30% correction loss (local kyphotic angle 22.6° before surgery, −1.5° immediately after surgery, and 7.0° at the final observation) [14]. However, there have been no reports about the changes in global alignment after anterior surgery for OVF. Our major clinical question in this study was whether sagittal imbalance following OVF could be improved by the anterior surgery or not. Therefore, the aim of this study was to report the correlation between local kyphotic changes and global spinal alignment after APSF in elderly OVF patients and to investigate the impact of global malalignment.

## 2. Materials and Methods

This multicenter retrospective cohort study was conducted at four institutions. Consecutive patients who underwent APSF for intra- or intervertebral instability after OVF were reviewed retrospectively.

The following were required of all patients eligible for participation in this retrospective study. (1) Osteoporotic vertebral fracture; (2) Intra- or intervertebral instability; (3) Neurologic deficit or severe back pain; and (4) Improvement of these symptoms in the supine position. Finally, the patients who were followed-up for at least 1 year were analyzed. Among them, patients with data of global spinal alignment before surgery and at final follow-up were included in the analysis. This study was approved by the institutional review board of our institution (approval no. 3170). The need to obtain informed consent was waived based on the retrospective design and anonymization of patient identifiers.

Patients’ clinical records were reviewed for demographic data, instability type, operation time (min), estimated blood loss (mL), performance status (PS, Common Toxicity Criteria, version 2.0), comorbidities, and perioperative complications. Bone mineral density (BMD) at the femoral neck was determined using dual-energy x-ray absorptiometry. Information on previous surgeries at the corpectomy site was obtained and divided into lumbar decompression, VP/BKP, and posterior instrumentation. The severity of pain was subjectively assessed by the patients on a visual analogue scale (VAS), which was based on the average level of back pain that the patient felt over the previous week. The VAS was measured before surgery and at final follow-up. The rate of minimal clinically important differences (MCID) was evaluated. MCID score for lumbar fusion surgery [15] was used (≥21 mm) because there have been no reports about MCID for OVF treatment. The fracture level was divided into thoracolumbar (T11–L2) and lumbar (L3–L5) regions.

Radiographic evaluation was performed via whole spine x-ray on all patients before surgery and at final follow-up and included analysis of sagittal alignment (sagittal vertical axis: SVA; pelvic incidence: PI; lumbar lordosis: LL; sacral slope: SS; pelvic tilt: PT; thoracic kyphosis: TK; T1 pelvic angle: TPA) and incidence of cage subsidence. Local kyphotic angle was defined as the angle between the inferior endplate of the vertebra above and the superior endplate of the vertebra below the fractured vertebra and was given a negative value in patients with kyphotic deformity. Intravertebral instability was defined as angular motion of the fractured vertebral body with intravertebral cleft between flexed and extended positions. Intervertebral instability was defined as a change in disc height of >2 mm with deformation of the vertebral body between flexed and extended positions.

### 2.1. Surgical Indications and Techniques

The patient was placed in a lateral position and a true lateral film was obtained with fluoroscopy. The affected vertebral body and the upper and lower discs were exposed per transthoracic retropleural or retroperitoneal approaches. After removal of discs above and below the affected vertebral body and the ligation or coagulation of segmental vessels, corpectomy was performed using a large osteotome. The cartilaginous endplate was carefully removed by a disc knife and ring curettage to prevent inadvertent endplate violation. The vertebral segment was reconstructed with an expandable titanium cage comprising rectangular footplates (X-Core2^®^; NuVasive, San Diego, CA, USA). Bone grafting was performed inside and outside of the cage using artificial tricalcium phosphate particles, resected vertebral body, and resected rib fragments. After position change, posterior percutaneous pedicle screw fixation (PPS) fixation was performed without decompression. The range of posterior fixation was unregulated and depended on the surgeon’s preference.

### 2.2. Statistical Analysis

Clinical outcomes were compared between postoperative SVA > 95 mm and ≤95 mm groups to investigate the impact of malalignment in patients who underwent this surgery [16]. In addition, baseline data, radiological parameters before surgery, and surgical complications were compared between SVA > 95 mm and ≤95 mm groups to investigate the factors related to SVA > 95 mm. Multiple logistic regression analysis was used to calculate odds ratios of variables for SVA > 95 mm. The model included age and variables with *p*-values < 0.10 in univariate analysis. The data on medication for osteoporosis including teriparatide, romosozumab, bisphosphonate, denosumab, and vitamin D within a month before index surgery were collected. We divided them into two groups in the analysis: bone-forming agents (teriparatide, romosozumab) and others.

Shapiro–Wilk tests were used to check normality assumptions for all parameters. The normality was confirmed in all continuous variables except for the VAS of back pain. The *t*-test (normality) or Mann–Whitney U test (non-normality) was used to compare continuous variables. The χ2 test or Fisher’s exact test was used for categorical variables. To establish whether significant differences existed in postoperative clinical or radiologic outcomes between the two group, a restricted maximum likelihood, mixed-model regression was used. Statistical test results were considered significant for values of *p* < 0.05. All *p*-values were two-sided. All analyses were performed using SAS version 9.4 (SAS Institute, Cary, NC, USA).

## 3. Results

A total of 72 patients were enrolled in this study. Two patients were lost to follow-up and one patient died two months after surgery due to pneumonitis. Fifteen patients were excluded due to insufficient radiological data. Finally, 54 patients were included in the analysis. Patients with a mean age of 76.3 years ± standard deviation 6.1 were followed-up for 25.3 months ± 12.6. Twelve patients (22%) had a history of thoracic or lumbar surgery. Regarding medication for osteoporosis, 32 patients (59%) were treated by teriparatide, 3 patients (6%) by romosozumab, 8 patients (15%) by bisphosphonate, 4 patients (7%) by denosumab, and 7 patients (13%) by only vitamin D. Mean operative time and estimated blood loss was 269.8 ± 79.8 min and 289.5 ± 289.5 mL, respectively. Regarding fixation range, 32 patients (59%) were one above and one below fixation. Adjacent vertebral fractures were observed in 11 patients (20%) after surgery.

Table 1 shows the radiological parameters before and after surgery. Local kyphosis, thoracic kyphosis, lumbar lordosis, SVA, TPA and PI-LL significantly improved at final follow-up compared with before surgery, although there was no improvement in PT and SS. Local kyphosis improved from −17.5 degrees to 4.1 degrees immediately after surgery but was −0.6 degrees at final follow-up with 22.4% of correction loss. SVA was improved by only 18.7 mm (from 111.8 mm to 93.1 mm).

Nineteen of the 54 patients (35%) showed global malalignment (SVA > 95 mm) postoperatively. Table 2 shows a comparison of baseline data, radiological parameters before surgery, and surgical complications between SVA > 95 mm and ≤95 mm groups. Adjacent vertebral fracture after surgery was significantly more frequent in the SVA > 95 mm group than in the SVA ≤ 95 mm group (37% vs. 11%, *p* = 0.038). TPA before surgery tended to be higher in the SVA > 95 mm group. Table 3 shows a comparison of clinical outcomes between SVA > 95 mm and ≤95 mm groups. VAS of back pain at final follow-up was significantly higher in patients with SVA > 95 mm than those in whom SVA was ≤95 mm (42.4 vs. 22.6, *p* = 0.015). Regarding the MCID, the better improvement was also observed in patients with SVA ≤ 95 mm (83% vs. 58%, *p* = 0.046). Multiple logistic regression showed a significantly increased odds ratio (OR) of adjacent vertebral fracture presence and TPA increase (OR = 4.76, 95% CI 1.10–20.58 and OR = 1.07, 1.00–1.14, respectively) (Table 4).

## 4. Discussion

This is the first study to reveal details about changes in sagittal balance following the minimally invasive procedure of corpectomy and reconstruction using an expandable cage with rectangular foot plates (APSF). Although there was 22.4% correction loss, local kyphotic changes using this system was 21.7°, which was better than the previous reports for APSF [17,18,19]. As well, Kanayama et al. reported that 80% of patients with OVF could be successfully treated using Kaneda instrumentation without the need for posterior reinforcement [20]. However, nearly 40% of correction loss was observed at the final follow-up. Suk et al. compared anterior-posterior surgery versus closing wedge osteotomy for kyphotic OVF and reported that the correction loss of anterior-posterior surgery was 27.3% with a mean blood loss of 2892 mL, whereas that of posterior closing wedge osteotomy was 10.8% with a mean blood loss of 1930 mL [21]. Posterior closing wedge osteotomy might offer better kyphosis correction. However, the procedure is technically demanding with more blood loss compared with the system in this study.

Although it is reported that anatomical and biomechanical restoration of vertebra is an advantage of anterior surgery resulting from the placement of anterior struts, our results indicated that restoration of sagittal alignment was not achieved by anterior surgery with 1–2 level posterior fixation in OVF patients. The parameters of SVA and TPA were used to evaluate sagittal spinal balance in this study. SVA increases with aging, and it is affected by movement of the hip and knee joint, such as “sway back” TPA, which combines information of SVA and PT and is a reliable indicator to address sagittal balance, including pelvic inclination [22]. TPA in this series was 33.2° preoperatively and 30.1° postoperatively. Thus, the improvement in TPA might not be significant. Ryan et al. demonstrated that TPA > 20° was the severe deformity threshold [23]. The main reason for this observation in our study was postoperative development of adjacent vertebral fracture. Low BMD, older age, an upper instrumented vertebra (UIV) level at the thoracolumbar spine, and a high preoperative SVA have been reported as risk factors for proximal junctional failure following surgical treatment for adult spinal deformity [24]. In the current series, BMD, medicine for osteoporosis, and level of surgery was not different between SVA > 95 mm and SVA ≤ 95 mm groups, probably because all the patients had comparatively severe osteoporosis. Posterior tethers and vertebral augmentation might be effective in preventing the failure of instrumentation, especially in patients with a high risk for proximal junctional kyphosis [25].

The relationship between PI and LL (PI-LL) is also considered an important parameter to evaluate sagittal spinal balance. Schwab et al. reported that SVA of 47 mm or more, PI-LL > 11° or more, and PT < 22° predicted severe disability (ODI > 40) [26]. Yamato et al. [31] described that the ideal LL angle can be determined using the equation ‘LL = 0.45 × PI + 31.8’. Inami et al. [27] reported that the optimum value of PI-LL is inconsistent, in that it depends on the individual PI. [28]. In this study, although PI-LL improved significantly (from 35.1° to 24.2°), the final PI-LL did not reach the ideal value. In addition, the preoperative decrease in SS did not change postoperatively, indicating absence of improvement of pelvic retroversion. If lumbar lordosis is restored by surgery, the retroverted sacrum must be improved to maintain spino-pelvic harmony. Otherwise, reciprocal changes in the thoracic spine might develop to maintain sagittal balance [29,30]. Our results showed an increase in TK from 26.8°to 32.8°, which concurred with the theory mentioned above. This reciprocal change might be one of the reasons SVA did not change significantly in the OVF patients in our study. Improvement of the retroverted sacrum requires extension of the hip joint, with the erector spinae and gluteus muscles playing an important role in this action. In aged OVF patients, weakness of these muscles is responsible for the pelvic retroversion [8,31]. The average age of patients in this study was 76.3 years; hence, although we did not measure muscle volumes in these patients, they might have had age-related muscle wasting and weakness. A retroverted pelvis can be managed surgically by osteotomy of the lower lumbar vertebra or long fixation involving the pelvis. However, these are extremely invasive surgeries and it is not clear whether such invasive correction surgery is necessary for aged OVF patients.

SVA changed from 111.8 mm to 93.1 mm, which, although a statistically significant change, might be an insufficient improvement to correct malalignment. Based on the classification of Scoliosis Research Society [16], SVA (>95 mm) was reported as a risk factor with the deterioration of QOL measures [32]. In the current study, the number of patients who acquired one or more level improvement of PS was 15/19 (78.9%) in SVA > 95 mm and 33/35 (94.3%) in SVA ≤ 95 mm groups, which although better in the SVA ≤ 95 mm group, was not significantly different. Postoperative VAS was better in the SVA ≤ 95 mm than the SVA > 95 mm group. As also reported by Hu et al. [5]. SVA correlated with back pain in this study, which significantly improved after surgery. However, age and preoperative comorbidities influence the complication rate in deformity surgery [33]. Thus, we thought that the strategy for aged OVF patients should differ from those in ASD patients to relieve pain and improve mobility. Our results also showed the significant improvement of JOA score and VAS even in the SVA > 95 mm group compared with those before surgery. It is not always necessary to restore sagittal imbalance in aged OVF patients to the same level as in young people, although the clinical results are worse in patients with SVA > 95 mm.

There are some limitations to this study. First, the number of patients was small because some patients were excluded due to lack of data from standing whole spine X-ray films before surgery because of intractable back pain. Second, due to the lack of apparatus, we did not take whole spine X-rays including the lower extremity. Hence, we could not evaluate knee and hip joint flexion, which might have been used to compensate for sagittal imbalance [34]. Despite these limitations, this is the first report describing the correlation between anterior spinal surgery and changes in sagittal alignment, which might contribute to preoperative planning in OVF patients. For further study, the prediction methods for postoperative sagittal balance are necessary, since this might contribute to decision-making in the surgical planning for OVF patients.

## 5. Conclusions

This study demonstrated the clinical and radiological outcomes of combined anterior–posterior procedures via a lateral corpectomy, vertebral reconstruction using an expandable cage with rectangular footplates and posterior percutaneous pedicle screw fixation. The procedure, which includes short segment fixation, did not improve global spinal alignment and pelvic retroversion. However, the procedure achieved significant reduction of local kyphosis and VAS of back pain. This indicated that the procedure is effective in elderly patients with severe back pain due to spinal deformity and instability caused by OVF despite the global spinal malalignment.

## Figures and Tables

**Table 1:** Comparison of local and global alignment pre- and postoperatively.

	Mean (SD)		*p*-Value
Local kyphosis			
Preop	−17.5	(19.2)	
Immediate postop	4.1	(13.1)	
Final	−0.6	(14.8)	
Δ (preop-final)	21.7	(13.3)	<0.001
Correction loss (%)	22.4	(42.5)	<0.001
TK			
Preop	26.8	(17.1)	
Final	32.8	(12.3)	
Δ (preop-final)	6.1	(15.2)	<0.001
LL			
Preop	14.6	(16.9)	
Final	25.5	(13.8)	
Δ (preop-final)	10.9	(14.7)	<0.001
SVA			
Preop	111.8	(45.6)	
Final	93.1	(46.6)	
Δ (preop-final)	18.7	(56.7)	0.018
PT			
Preop	28.4	(7.9)	
Final	27	(8.2)	
Δ (preop-final)	1.4	(8)	0.209
SS			
Preop	21.5	9.8	
Final	22.8	10.0	
Δ (preop-final)	1.2	7.4	0.229
TPA			
Preop	33.2	(10.4)	
Final	30.1	(9.3)	
Δ (preop-final)	3.1	(9.5)	0.019
PI-LL			
Preop	35.1	(17.7)	
Final	24.2	(14.4)	
Δ (preop-final)	10.9	(14.7)	<0.001

SD, standard deviation; TK, Thoracic kyphosis; LL, Lumbar lordosis; SVA, Sagittal vertical axis; PT, Pelvic tilt; SS, sacral slope; TPA, T1 Pelvic Angle; PI-LL, Pelvic incidence- Lumbar lordosis.

**Table 2:** Comparison between SVA > 95 mm and ≤95 mm groups by univariate analysis.

	SVA > 95 mm (*n* = 19)		SVA ≤ 95 mm (*n* = 35)		*p*-Value
	Mean or N (SD or %)		Mean or N (SD or %)		
Age	76.9	(5.8)	76	(6.2)	0.577
Gender	13	(68)	26	(74)	0.646
Follow-up period (months)	28.9	(13.4)	23.3	(11.8)	0.121
BMD (T-score)	−2.4	(0.5)	−2.1	(0.9)	0.251
Medication for osteoporosis					
Teriparatide/Romosozumab	13	(68)	22	(63)	0.683
Previous surgery					
Lumbar decompression	1	(5)	4	(11)	
Vertebral augmentation	1	(5)	1	(3)	
Posterior instrumentation	1	(5)	4	(11)	0.781
Level					
Thoracolumbar	10	(53)	17	(49)	
Lumbar	9	(47)	18	(51)	1.000
Proximal fixation range					
1	11	(58)	21	(60)	
>1	8	(42)	14	(40)	1.000
Distal fixation range					
1	13	(68)	24	(69)	
>1	6	(32)	11	(31)	1.000
Adjacent vertebral fracture	7	(37)	4	(11)	0.038
Infection	1	(5)	1	(3)	1.000
Reoperation	3	(16)	2	(6)	0.332
Cage subsidence	9	(47)	15	(43)	0.750
Local kyphosis preop	−21.7	(15.3)	−15.3	(20.8)	0.248
Local kyphosis at final FU	−2.2	(12)	0.3	(16.2)	0.549
LL preop	9.8	(17.8)	17.2	(16)	0.126
PT preop	30.6	(7.7)	27.1	(7.9)	0.127
PI preop	52.1	(10.7)	48.4	(9.5)	0.190
SVA preop	122.4	(45.4)	106.1	(45.4)	0.217
TK preop	21.5	(16.6)	29.6	(17)	0.100
TPA preop	36.9	(10.6)	31.2	(9.9)	0.052

SD, standard deviation; BMD, Bone marrow density; TK, Thoracic kyphosis; LL, Lumbar lordosis; SVA, Sagittal vertical axis; PT, Pelvic tilt; SS, sacral slope; TPA, T1 Pelvic Angle; PI-LL, Pelvic incidence- Lumbar lordosis.

**Table 3:** Comparison of clinical outcomes between SVA > 95 mm and ≤95 mm groups.

	SVA > 95 mm (n = 19)		SVA ≤ 95 mm (n = 35)		*p*-Value
	Mean or N (SD or %)		Mean or N (SD or %)		
PS improvement (N)	15	(79)	33	(94)	0.087
JOA score					
Preop	10.9	(5.1)	9.5	(4.8)	0.311
Final	19.2 *	(5.4)	20.5 *	(4.7)	0.361
Improvement ratio	46.1	(19.8)	54.8	(28.7)	0.248
VAS of back pain					
Preop	73.7	(17.8)	77.3	(23)	0.301
Final	42.4 *	(28.7)	22.6 *	(23)	0.015
Δ (preop-final)	31.4	(23.2)	54.7	(30.8)	0.008
MCID (≥21 mm)	11	(58)	29	(83)	0.046

SD, standard deviation; PS, Performance Status; JOA score, The Japanese Orthopaedic Association score; MCID, minimal clinically important difference. * There were significant differences between preop and final scores of JOA score and VAS of back pain.

**Table 4:** Adjusted odds ratio for SVA > 95 mm at final follow-up.

	Adjusted OR *	95% CI		*p*-Value
TPA preop (per 1 degree)	1.07	1.00	1.14	0.047
Adjacent vertebral fracture	4.76	1.10	20.58	0.037

TPA, T1 Pelvic Angle; OR, odds ratio. * The odds ratio was adjusted for age, preoperative TPA and adjacent vertebral fracture.
